# Structural modeling of Na_v_1.5 pore domain in closed state

**DOI:** 10.52601/bpr.2021.200021

**Published:** 2021-08-31

**Authors:** Xiaofeng Ji, Yanzhao Huang, Jun Sheng

**Affiliations:** 1 Yellow Sea Fisheries Research Institute, Chinese Academy of Fishery Sciences, Qingdao 266071, Shandong, China; 2 School of Physics, Huazhong University of Science and Technology, Wuhan 430074, China

**Keywords:** Na_v_1.5 ion channel, Closed state, Homology modeling, Direct coupling analysis, Local anesthetics, Drug binding

## Abstract

The voltage-dependent cardiac sodium channel plays a key role in cardiac excitability and conduction and it is the drug target of medically important. However, its atomic- resolution structure is still lack. Here, we report a modeled structure of Na_v_1.5 pore domain in closed state. The structure was constructed by Rosetta-membrane homology modeling method based on the template of eukaryotic Na_v_ channel Na_v_PaS and selected by energy and direct coupling analysis (DCA). Moreover, this structure was optimized through molecular dynamical simulation in the lipid membrane bilayer. Finally, to validate the constructed model, the binding energy and binding sites of closed-state local anesthetics (LAs) in the modeled structure were computed by the MM-GBSA method and the results are in agreement with experiments. The modeled structure of Na_v_1.5 pore domain in closed state may be useful to explore molecular mechanism of a state-dependent drug binding and helpful for new drug development.

## INTRODUCTION

Voltage-gated sodium (Na_v_) channels are a class of trans-membrane ion channels that serve as channels for sodium ions to pass through the cell. Na_v_1.5 is a cardiac isoform of Na_v_ channel and is the key target of pharmaceutical drugs. Knowing the tertiary structure of Na_v_1.5 will facilitate to understand the molecular mechanism of the drugs. However, its three-dimensional (3D) structure is still unknown and so structural modeling methods have been predominantly employed to fill this gap (O’Reilly *et al*. 2012; Payandeh *et al*. 2011; Pless *et al*. 2011).

In the early stage, due to the lacking of experimental structures of Na_v_ channel, all modelled structures were modeled by using potassium channels as templates (Lipkind and Fozzard 2000; O’Reilly *et al*. 2012). The structures of bacterial Na_v_ channels, including Na_v_Ab (Payandeh *et al*. 2011, 2012), Na_v_Ms (Bagneris *et al*. 2014; McCusker *et al*. 2012), and Na_v_Rh (Zhang *et al*. 2012), were successively resolved and they revealed that the Na_v_ channels were formed by four domains (DI to DIV) and each domain consisted of six transmembrane segments. In each domain, voltage sensor domain (VSD) was composed by the first four transmembrane segments (S1 to S4) and the ion conducting pore domain (PD) was formed by the other two segments (S5 and S6), with the pore loop (P-loop) between them. Based on these structures, Na_v_1.5 models were also reported (Ahmed *et al*. 2017; Moreau *et al*. 2015; Poulin *et al*. 2014; Wang *et al*. 2015; Xia *et al*. 2013). Recently, we also constructed an open-state structure of Na_v_1.5 by using the bacterial Na_v_Ms as template (Ji *et al*. 2018). As we all know, the bacterial Na_v_ channel shares similar overall architecture with eukaryotic one but differs in that the former is homo-tetramer while the latter is non-homo-tetramer. Recently, the first structure of eukaryotic Na_v_ channel (Na_v_PaS) at near-atomic resolution has been resolved (Shen *et al*. 2017) and this makes it possible to build structures of eukaryotic Nav channels with higher precision.

Homology modeling methods have been widely used to construct 3D models of proteins from their amino acid sequences (Korkosh *et al*. 2014; Lafita *et al*. 2018). These methods were based on the similarity of the sequence of the target protein to other proteins whose 3D structures were known. The Rosetta membrane homology modeling method (Sidhartha *et al*. 2010; Subbotina *et al*. 2010) were used for constructing 3D models of membrane proteins. It searched for fragments with similar sequences in 3D structure databases and could predict membrane protein structure with reasonable accuracy (Subbotina *et al*. 2010).

In the structural modeling, information of residue–residue contacts will facilitate to find out the best conformation from the modeled decoys by combining with energy score, but experimental information of residue–residue contacts is usually unknown in advance. Therefore, the prediction of the contacts from sequences will be very helpful. The direct coupling analysis (DCA) of co-evolutionary residues has been shown to be an efficient method to do this (Jarzynski 2012; Lunt *et al*. 2010; Morcos *et al*. 2014; Morcos *et al*. 2011; Weigt *et al*. 2009). DCA gives a score for each pair of residues in an amino-acid sequence or between two sequences to evaluate if the two residues can form contact.

In this work, we will model closed-state structures of Na_v_1.5 pore domain (PD) based on the template of eukaryotic Na_v_ channel Na_v_PaS (Shen *et al*. 2017) and apply RosettaMP energy and DCA to select the rational structures of monomers and PD. We will validate the modeled structures by docking closed-state local anesthetics (LAs) to it and compare their binding energies and binding sites with previous experimental results.

## RESULTS AND DISCUSSION

### EC-score calculation for the sodium channel membrane proteins with known structures

**Figure 1:**
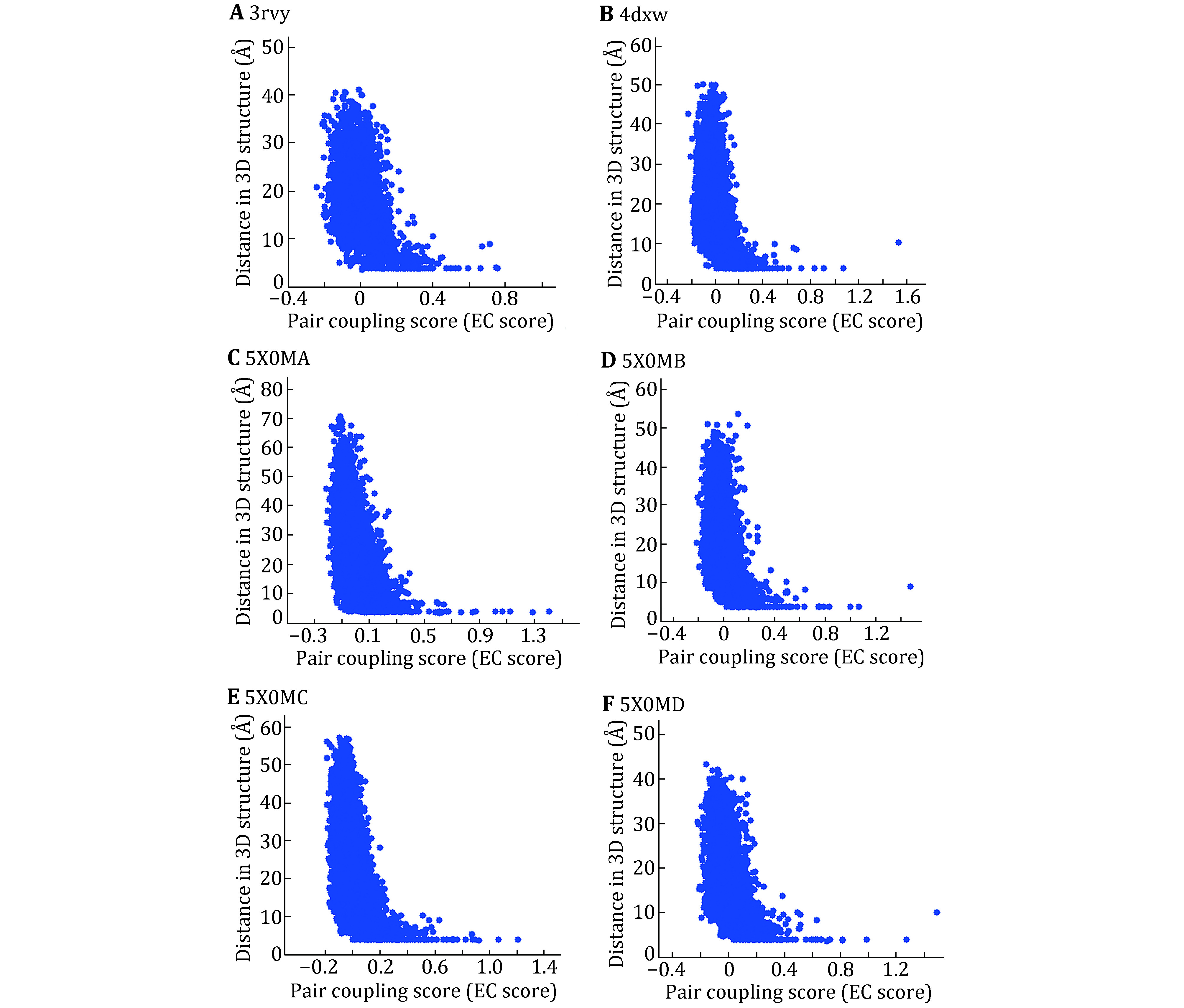
Maps of EC score and residue–residue distances for the three Na_v_ channels with known tertiary structure. 3rvy (**A**), 4dxw (**B**) and the four domains of 5x0m (**C**–**F**). *X* axis is EC score and *Y* axis is residue–residue distance in the 3D structure

We first investigate whether the co-evolutionary residues of intra- and inter-domains of Na_v_ channels do exist in the tertiary structure by assessing blinded predictions of residue co-evolution against the determined structures Na_v_Ab (PDB ID: 3rvy), Na_v_PaS (PDB ID: 5x0m) and Na_v_Rh (PDB ID: 4dxw). The results of the multiple sequence alignments were listed in supplementary Table S1. For each pair of residues within or across the domains, EVcouplings calculates an EC score for it, which describes the degree of co-evolution of the two residues. Because the four domains of Na_v_Ab and Na_v_Rh have the same sequence, but the four domains of Na_v_PaS are different, we only calculate the EC score and residue–residue distances of one domain for Na_v_Ab and Na_v_Rh, and the EC score and residue–residue distances for Na_v_PaS. Figure 1 shows the correlation between EC scores and residue–residue distances of intra-domains. Here we define that a pair of residues with an 8 Å minimum atom distance between them are in contact. From Fig. 1, we can find that the distance of two residues is usually less than 8 Å when the EC score is large than 0.2. Therefore, if the EC score of two residues is more than 0.2, the two residues are considered to form contact in the 3D structure.

Based on this threshold (0.2), the intra- and inter-contact pairs of the domains are predicted and the number of predicted contact pairs is labeled as CA_DCA. Number of contact pairs in 3D structure is labeled as CA_Str. To access the accuracy rate of the predicted contacts, we investigated the coincidence of the predicted contact pairs and those in 3D structure. Table 1 shows that among 52 predicted contact pairs in single domain, there are 32 contact pairs that do have contacts in the 3D structure of Na_v_Ab and the rate of correctly predicted is nearly 62%. In particular, among the predicted contact pairs with top ten EC scores, there are eight pairs in the 3D structure. Similar to Na_v_Ab, the accuracies for Na_v_Rh and Na_v_PaS are more than 50% and for 5x0mD it is even more than 69%. Because among the three Na_v_ channels with known structures, only the domains of Na_v_PaS have distinct conformations, it was chosen to be the evaluation protein to access whether the EC scores can be used to predict contact

**Table 1:** Intra-EC pairs for the three Na_v_ channels with known tertiary structure

Name	Structure (PDB ID)	CA_Str	CA_DCA	N_Coin	N/DCA (%)	N_T10
Na_v_Ab	3rvy	539	52	32	61.538	8
Na_v_Rh	4dxw	603	50	31	62.000	8
Na_v_PaS	5x0mA	1009	94	51	54.255	7
	5x0mB	661	54	28	51.851	6
	5x0mC	860	80	45	56.250	6
	5x0mD	675	52	36	69.230	7
“CA_Str” is the number of contact pairs in the tertiary structure; “CA_DCA” is the number of predicted pairs; “N_Coin” is the number that the predicted contact pairs do exist in 3D structure; “N/DCA” (N_Coin/CA_DCA) is the precision of prediction. “N_T10” is the number that the predicted contact pairs of top 10 ECs that do exist in 3D structure						

residues between protein domains. The inter-EC pairs for Na_v_PaS are listed in Table 2. Compared to intra-contacts, the accuracy of inter-contact prediction is very lower. For the adjacent domains (AB, BC, CD and AD), the precision is about 10%. These results demonstrate that the EC scores can be used to determine contacts in one of the domains of Na_v_ channels but are not good enough for inter-contacts between the domains. However, the latter can also be considered as a reference since we can see from Table 2 that the precisions of the predicted contacts between the adjacent domains are much higher than those of nonadjacent domains. By the way, these results also indicate that the formation of the tetramers of Na_v_ channels may not be determined mainly by the specific interactions of the co-evolved residues between the domains but by other interactions.

**Table 2:** Inter-EC pairs for Na_v_PaS

Name	Structure (PDB ID)	CA_str	CA_DCA	N_Coin	N/DCA (%)	N_T10
Na_v_PaS	5x0mAB	50	36	5	13.89	1
	5x0mAC	2	38	1	2.63	0
	5x0mAD	54	40	3	7.50	0
	5x0mBC	64	38	4	10.53	1
	5x0mBD	1	52	0	0	0
	5x0mCD	43	35	4	11.43	0

### Structural modeling and selection of Na_v_1.5 domains

Using sequences of the four domains of Na_v_1.5 as the input, we searched the database of Protein Data Bank (PDB, www.rcsb.org.com) using BlastP. From the search results, we found that compared to other four bacterial Na_v_ channels (Na_v_Ab, Na_v_Rh and Na_v_Ms, Na_v_PaS), all of the four target sequences had the highest sequence identities (48%) with the corresponding domains of Na_v_PaS. Meanwhile, Na_v_PaS was the sole eukaryotic sodium ion channel with known tertiary structure by far and it had a closed pore. Hence, the structure of Na_v_PaS was chosen as the template structure and the ROSETTA membrane-homology modeling method was used to model the PD structure of Na_v_1.5 channel in closed state. The four target sequences were aligned to corresponding sequences of Na_v_PaS by HHpred program with the parameters as system default (E-value cutoff 1 × 10^−3^, minimum coverage of multiple sequence alignment hits 20%, realignment threshold 0.3). The alignment results (supplementary Fig. S1) show that the insertion and deletion are mainly in P-segments but not any in S5 and S6. For each domain, 10,000 models were generated. Then, we calculated their energies by using RosetaMP and the distances between any two residues. We also calculated the EC scores of each residue pairs by using EVcouplings. For the four domains, 1939, 3923, 3390 and 3597 homologous sequences were used to generate the multiple sequence alignment to calculate EC scores, respectively. Table 3 shows the intra-EC pairs for the four domains of Na_v_1.5. From Table 3, we can find that for domain 1, structure domain1_5x0mS_0177 and domain1_5x0mS_0467 are the top two structures with the highest energy score and precision of contact prediction, *i*.*e*. these two structures not only have the lowest energy, but also have the highest consistency of the residue–residue contacts with the DCA prediction. Hence these two structures were selected as the modeled structures of domain 1. Similar to this procedure, the modeled structures for other three domains were selected (the bold in Table 3). Meanwhile, we can find out that these selected structures have the lowest energy as well as higher precision of contact prediction. The Ramachandran plots of the selected models also show that the dihedral angles of all residues are located in the allowed regions (supplementary Fig. S2). The helices S5 and S6 of the selected models are nearly the same and the differences occur in the region of P-segments (supplementary Fig. S3).

**Table 3:** Candidates for structures of the four domains of Na_v_1.5

Structure	Energy	CA_str	CA_DCA	N_Coin	N/DCA (%)	N_T10
DI structure						
**domain1_5x0mS_0177**	**−259.869**	**1405**	**63**	**44**	**69.841**	**4**
**domain1_5x0mS_0467**	**−257.737**	**1450**	**63**	**43**	**68.253**	**3**
domain1_5x0mS_0792	−257.319	1431	63	43	68.253	4
domain1_5x0mS_0264	−257.180	1486	63	40	63.492	3
domain1_5x0mS_0772	−254.850	1439	63	42	66.666	4
domain1_5x0mS_0062	−251.525	1326	63	41	65.079	3
domain1_5x0mS_0972	−250.669	1450	63	42	66.666	3
domain1_5x0mS_0982	−250.593	1417	63	44	69.841	4
domain1_5x0mS_0784	−249.046	1365	63	41	65.079	2
domain1_5x0mS_0490	−248.589	1372	63	39	61.904	3
DII structure						
**domain2_5x0mS_0322**	**−201.976**	**917**	**48**	**33**	**68.750**	**8**
**domain2_5x0mS_0980**	**−195.486**	**917**	**48**	**34**	**70.833**	**8**
domain2_5x0mS_0393	−194.378	910	48	33	68.750	8
domain2_5x0mS_0886	−191.752	898	48	33	68.750	9
domain2_5x0mS_0345	−191.497	874	48	31	64.583	8
domain2_5x0mS_0279	−191.085	876	48	31	64.583	8
domain2_5x0mS_0715	−190.996	903	48	32	66.666	9
domain2_5x0mS_0442	−190.847	903	48	32	66.666	8
domain2_5x0mS_0285	−190.438	903	48	33	68.750	8
domain2_5x0mS_0561	−190.080	879	48	31	64.583	8
DIII structure						
**domain3_5x0mS_0433**	**−233.286**	**1289**	**69**	**50**	**72.463**	**6**
**domain3_5x0mS_0501**	**−235.480**	**1277**	**69**	**47**	**68.115**	**6**
domain3_5x0mS_0084	−232.065	1287	69	48	69.565	6
domain3_5x0mS_0453	−230.977	1216	69	50	72.463	6
domain3_5x0mS_0270	−228.602	1253	69	50	72.463	6
domain3_5x0mS_0259	−228.583	1231	69	50	72.463	6
domain3_5x0mS_0034	−227.736	1270	69	50	72.463	6
domain3_5x0mS_0716	−223.270	1253	69	48	69.565	6
domain3_5x0mS_0250	−220.025	1277	69	50	72.463	6
domain3_5x0mS_0688	−219.165	1243	69	50	72.463	6
DIV structure						
**domain4_5x0mS_0219**	**−193.494**	**968**	**51**	**39**	**76.470**	**9**
**domain4_5x0mS_0838**	**−188.711**	**942**	**51**	**38**	**74.509**	**9**
domain4_5x0mS_0226	−186.353	930	51	38	74.509	9
domain4_5x0mS_0120	−185.934	936	51	40	78.431	9
domain4_5x0mS_0531	−185.682	904	51	39	76.470	9
domain4_5x0mS_0878	−185.430	943	51	39	76.470	9
domain4_5x0mS_0087	−184.725	937	51	40	78.431	9
domain4_5x0mS_0430	−184.024	916	51	38	74.509	9
domain4_5x0mS_0011	−183.128	892	51	36	70.588	9
domain4_5x0mS_0318	−182.277	925	51	38	74.509	9

Finally, 16 PD models were generated by using the selected models of the four domains and scored by RosettaMP. From Table 4, we can see that the RosettaMP energy of closed10 is the lowest. It is noted that the N/DCA value of closed10 is also the highest although its absolute value is low. Therefore, the closed10 is selected for subsequent research (the bold in Table 4).

**Table 4:** Energy and inter-domain EC pairs of 16 PD models

	Structure	Energy	CA_str	CA_DCA	N_Coin	N/DCA (%)
closed1	0117_0322_0433_0219	−135.204	265	481	11	2.287
closed2	0117_0322_0433_0838	−494.138	269	481	12	2.495
closed3	0117_0322_0501_0219	−220.395	270	481	11	2.287
closed4	0117_0322_0501_0838	−308.781	277	481	12	2.495
closed5	0117_0980_0433_0219	−169.464	236	481	7	1.455
closed6	0117_0980_0433_0838	−184.004	233	481	9	1.871
closed7	0117_0980_0501_0219	−258.261	239	481	10	2.079
closed8	0117_0980_0501_0838	−271.122	245	481	11	2.287
closed9	0467_0322_0433_0219	−496.215	295	481	15	3.119
**closed10**	**0467_0322_0433_0838**	**−524.288**	**301**	**481**	**18**	**3.742**
closed11	0467_0322_0501_0219	−215.505	298	481	14	2.911
closed12	0467_0322_0501_0838	−281.421	313	481	16	3.326
closed13	0467_0980_0433_0219	−348.618	268	481	12	2.495
closed14	0467_0980_0433_0838	−524.150	275	481	15	3.119
closed15	0467_0980_0501_0219	−497.223	267	481	14	2.911
closed16	0467_0980_0501_0838	−522.211	283	481	16	3.326
“Structure” is the assembling structure from each of the four domains selected in Table 3; “CA_str” is the numbers of contact pairs in the tertiary structure; “CA_DCA” is the number of predicted pairs that with EC value larger than 0.2; “N_Coin” is the number that the predicted contact pairs do exist in 3D structure; “N/DCA” is the precision of prediction						

**Figure 2:**
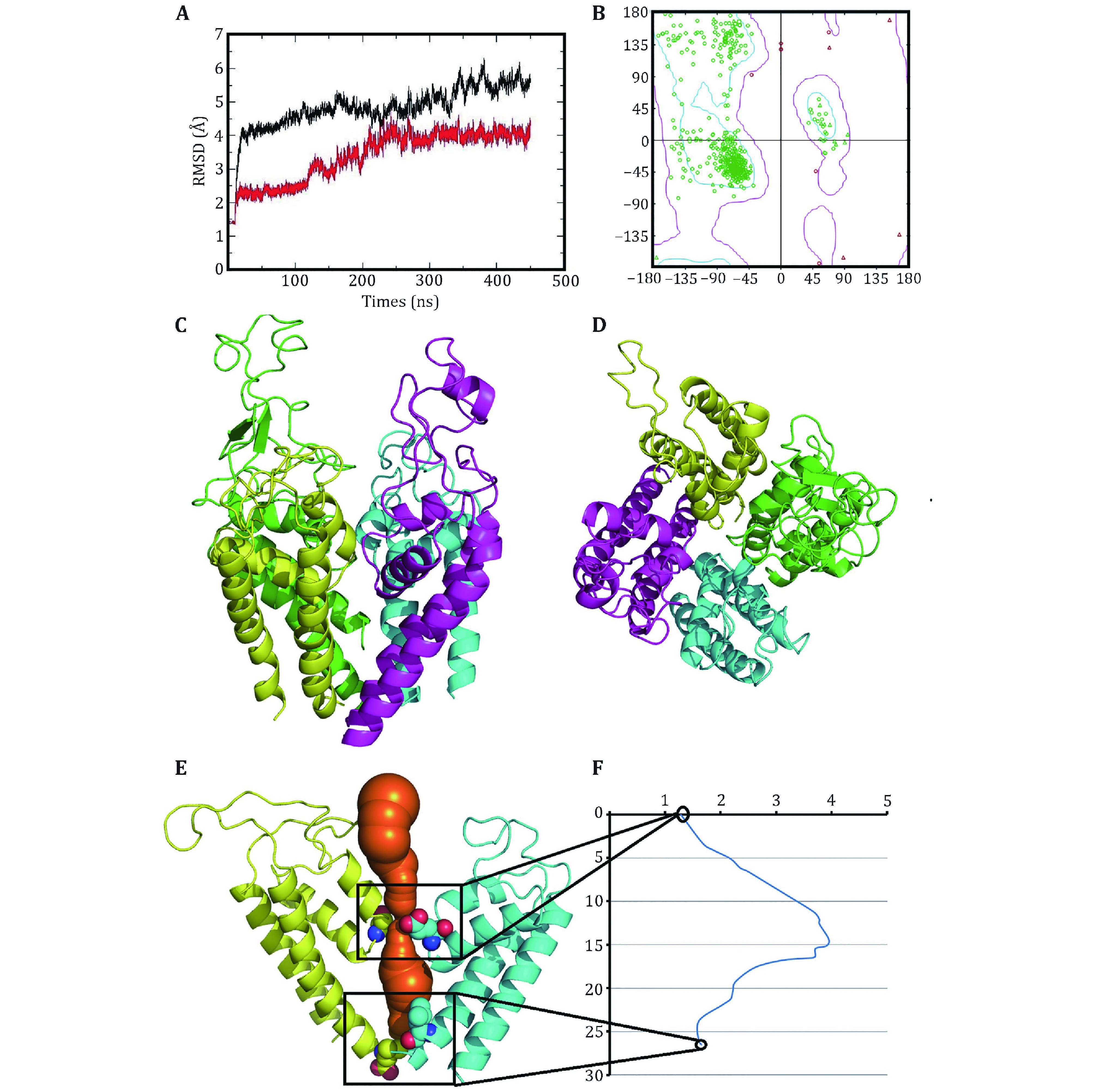
Optimized structure of the closed10. **A** The changes of the RMSDs of Cα atoms of the closed10 with (black line) and without (red line) outer membrane P-loops during 450-ns MD simulation. **B** Ramachandran plot of the optimized structure. The side (**C**) and top (**D**) views of the optimized structure, generated by pymol (Lilkova *et al*. 2015) and colored by chain (green, cyan, purple and yellow for D1−D4). The channel (in orange) (**E**) and its radius versus distance along the channel direction (**F**)

**Figure 3:**
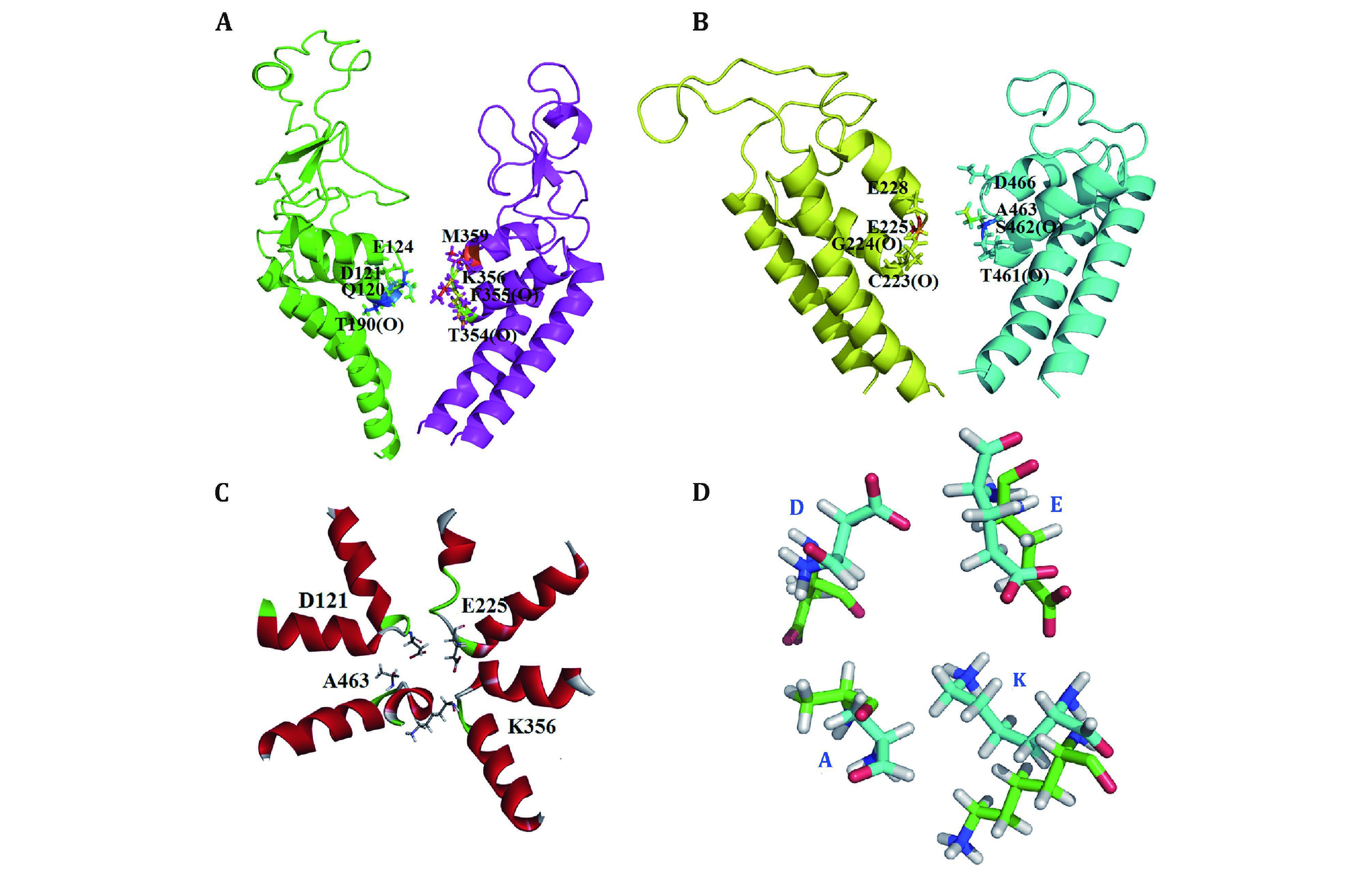
The selectivity filter (SF) of the model closed10. **A**, **B** The SF residues D121/E225/K356/A463 with their two preceding residues and the residues that constitute the negative ring above the SF vestibule. All these residues are represented in stick. **C** SF top view is shown. **D** SF structural variations of closed10 (green) and Na_v_PaS (cyan)

To destroy atom clashes and tensions, the close10 is further optimized by a 450-ns MD simulation and finally reaches an equilibrated structure with a fluctuated outer-membrane P-loops (Fig. 2A and supplementary Fig. S4). The Ramachandran plot also shows that the dihedral angles of almost all residues in the equilibrated structure are located in the allowed regions (Fig.2B). Figures 2C and 2D show the equilibrated structure from different views. Figures 2E and 2F show the channel and its radius versus distance along the channel direction for the equilibrated structure, respectively. They show that the diameter of the gate region is about 3.48 Å, which is much smaller than that of sodium ions (roughly 7.8 Å) and so sodium ions cannot pass through the channel in this case. This means that the constructed model is in closed state. Furthermore, Figures 3A and 3B show the selective filter (SF) of the close10 formed by the side groups of residues D121/E225/K356/A463 and the carbonyl oxygen atoms of their two preceding residues.

The outer negative ring that guards the entrance to the SF vestibule is formed by E124/E228/M359/D466. Compared to Na_v_PaS, the differences are mainly in the orientation of residue K356 and D121 (Fig. 3C and 3D). Aligned the structure of the closed10 without outer membrane to its open state that we had constructed before by using TM-align (Zhang and Skolnick 2005), the result shows that the RMSD is 3.77 and the TM-score is 0.41606. Furthermore, in order to check the stability of the modelled structure, we calculated the diameter of the gate region and the all atoms RMSF during the simulation. For diameter calculation, the larger distance of D121-K356 and E225-A463 is defined as the diameter of the gate region of the corresponding frame. The results show that during the simulation, the diameter of the gate region is in the range of 4.19 to 1.21 (supplementary Fig. S5A), which is smaller than that of sodium ions, *i*.*e*. the door is closed during the simulations. RMSF values were then calculated to analyze the fluctuations of all residues (supplementary Fig. S5B). In supplementary Fig. S5B, the higher values are found in regions of loops and the connection areas of the domains. This means that the positions of residues in regions of S5, S6 and P-loops in the four domains are almost unchanged.

### Structure validation

Previous studies show that the inner vestibule of Na_v_1.5 PD is the binding pocket of open-state and closed-state LA drugs (Lipkind and M 2005) and the residues L214 (L1462 in Na_v_1.5α) in S6 of D3, and F512 (F1760 in Na_v_1.5α) and Y519 (Y1767 in Na_v_1.5α) in S6 of D4 are parts of their binding sites. The closed-state LAs include Pilsicainide, Bisphenol A and Mexiletine, and the binding strength of them has been measured by experiments. Therefore, this information can be used to validate the closed10 model by finding the binding sites and binding energies of the closed-state LAs with the closed10.

**Figure 4:**
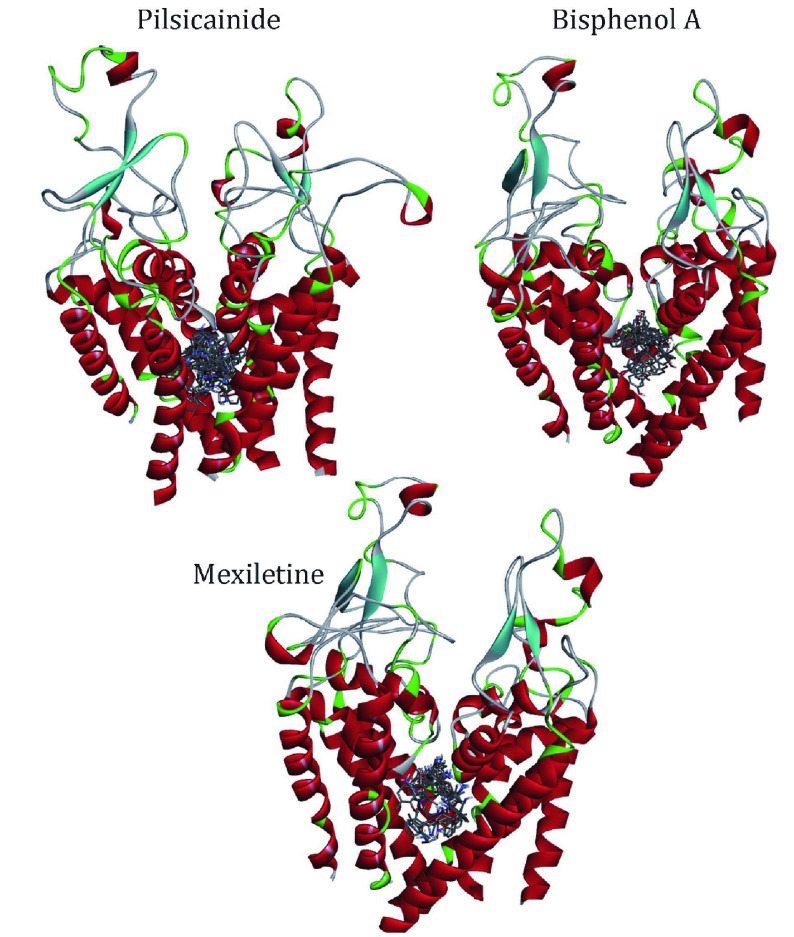
The relative position of docking conformations of LAs with the closed10. The plot is generated by DS Visualizer 4.2, protein structures are shown in cartoon and drugs are shown in sticks

We docked the three closed-state LAs to the closed10. Three independent dockings were made and the conformations of LAs were clustered with the cutoff of 2 Å (Table 5). Mapping all of these conformations to the closed10 (Fig. 4), it can be found that all of them are located within the cavity enclosed by S6 segments. This result is consistent with previous reports. For Pilsicainide, Bisphenol A and Mexiletine, the clusters C1, C4 and C2 have the lowest average binding energy respectively, and the conformations with the lowest binding energy are also in these clusters. These three lowest energy conformations are chosen to select their complexes with the closed10, respectively.

**Table 5:** Cluster results of conformations of three independent dockings for Pilsicainide, Bisphenol A and Mexiletine

	Pilsicainide				Bisphenol A				Mexiletine		
	Nc	Ave. (kcal/mol)	Min. (kcal/mol)		Nc	Ave. (kcal/mol)	Min. (kcal/mol)		Nc	Ave. (kcal/mol)	Min. (kcal/mol)
C1	5	−120.39	−122.58		5	−120.51	−121.24		5	−119.79	−120.01
C2	4	−120.05	−122.05		4	−120.07	−120.64		4	−119.84	−120.81
C3	3	−119.35	−120.44		3	−120.55	−121.25		3	−120.38	−120.47
C4	3	−119.87	−120.48		3	−121.49	−121.98		3	−120.17	−120.21
C5	3	−119.44	−120.06		3	−120.72	−120.93		3	−120.70	−120.78
C6	3	−119.40	−122.64		3	−120.24	−121.80		2	−120.42	−120.57
C7	1	−119.74	−119.74		2	−120.13	−120.55		1	−119.74	−119.74
C8					2	−119.52	−119.94		1	−119.86	−119.86
C9					1	−121.41	−121.41		1	−119.50	−119.50
C10									1	−119.09	−119.09
“C1”–“C10” demonstrate the 10 clusters, “Nc” is the number of conformations, “Ave.” is the average energy of all the conformations in the cluster, “Min.” is the lowest energy of all conformations of the cluster											

**Figure 5:**
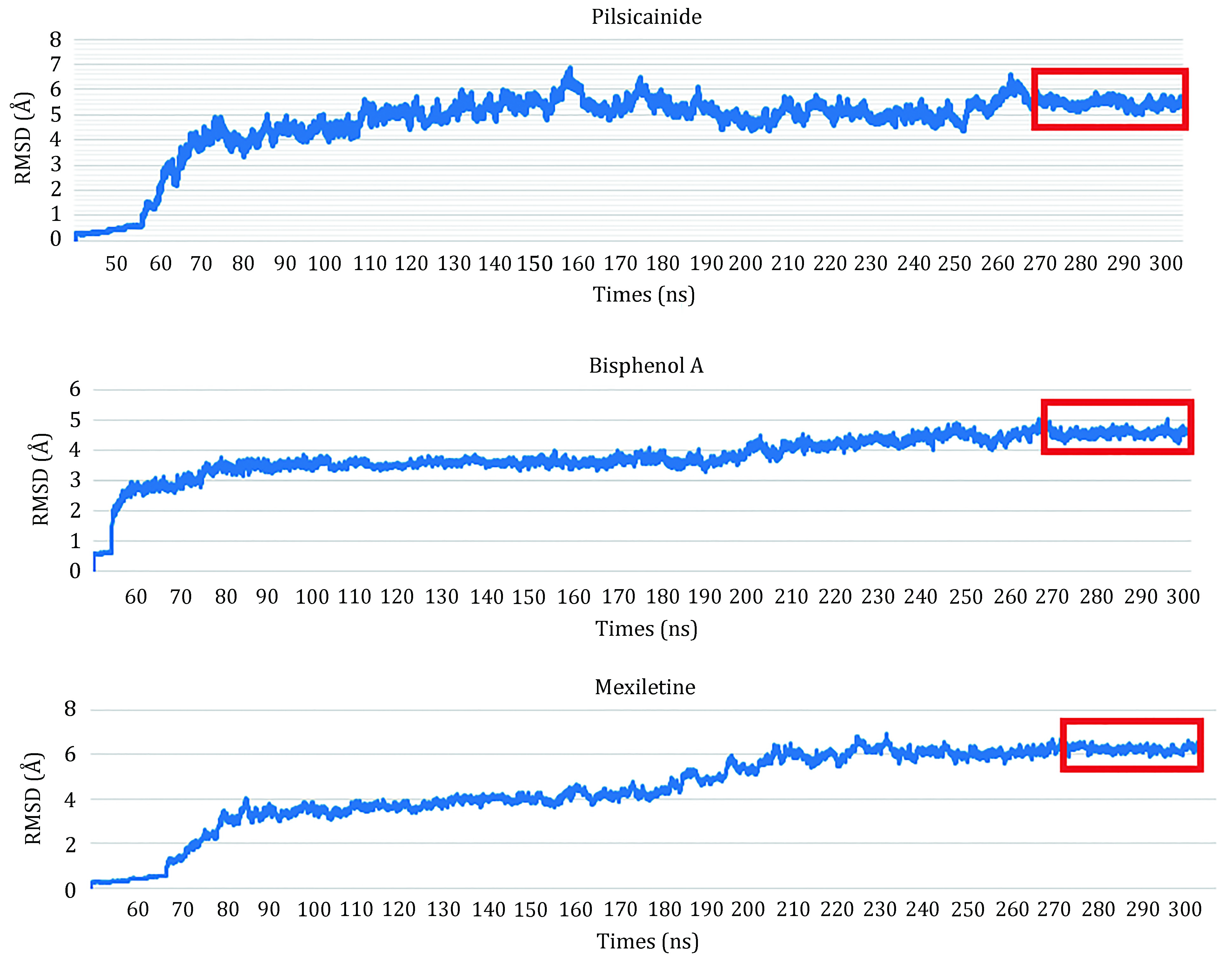
RMSDs for the complexes of Na_v_1.5_Las. The trajectories in the red rectangles are used to calculate binding energies

In order to detect the ability of our model to discriminate strong from weak closed-state LAs, we calculated the binding energies of LA-closed10 complexes. For each complex, 300-ns MD simulation was done and the last 30 ns (3 × 10^3^ frames) was used to calculate the binding energy (Fig. 5). In order to clarify the dynamic stability of these structures, RMSD values were obtained using the initial structures as templates for ligands (supplementary Fig. S6). The L_RMSD plots show that the RMSD values of the atoms (in nofit) with respect to the initial structures increased for 150 ns. After that, they remained fluctuated around 1.7–2 Å until the end of the simulation. Thus, the trajectories of the MD simulations of these structures were reliable.

The binding energies calculated by MM-GBSA are summarized in Table 6. It can be found that polar solvation plays positive role while no-polar solvation plays negative role for interaction of LAs and Na_v_1.5, *i*.*e*. van der Waals, electrostatic and accessible surface area facilitate the binding of LAs with Na_v_1.5 while the polar solvation destabilizes their binding. Totally, the closed10 binds more tightly with Mexiletine than the other two Las, and binds most loosely with Pilsicainide. This result is consistent with the previous reports about *K*_d_ values of these three LAs (Li *et al*. 1999; Reilly *et al*. 2012; Pless *et al*. 2011).

**Table 6:** Binding energies of LA_closed10 complexes

	Energies (kcal/mol)		
	Na_v_1.5_Pilsicainide	Na_v_1.5_Bisphenol A	Na_v_1.5_Mexiletine
G_gas_			
Electrostatic	−14.38 ± 0.76	−28.96 ± 0.41	−23.30 ± 1.52
Van der Waals	−5.52 ± 0.57	−1.75 ± 0.14	−14.36 ± 0.15
G_solv_			
GB	19.41 ± 0.61	9.8324 ± 0.67	14.83 ± 0.39
SURF	−1.79 ± 0.55	−3.87 ± 0.03	−3.85 ± 0.17
Binding energy	−2.28 ± 1.86	−24.75 ± 0.23	−26.637 ± 1.94
Results are presented as average ± standard error.			

**Figure 6:**
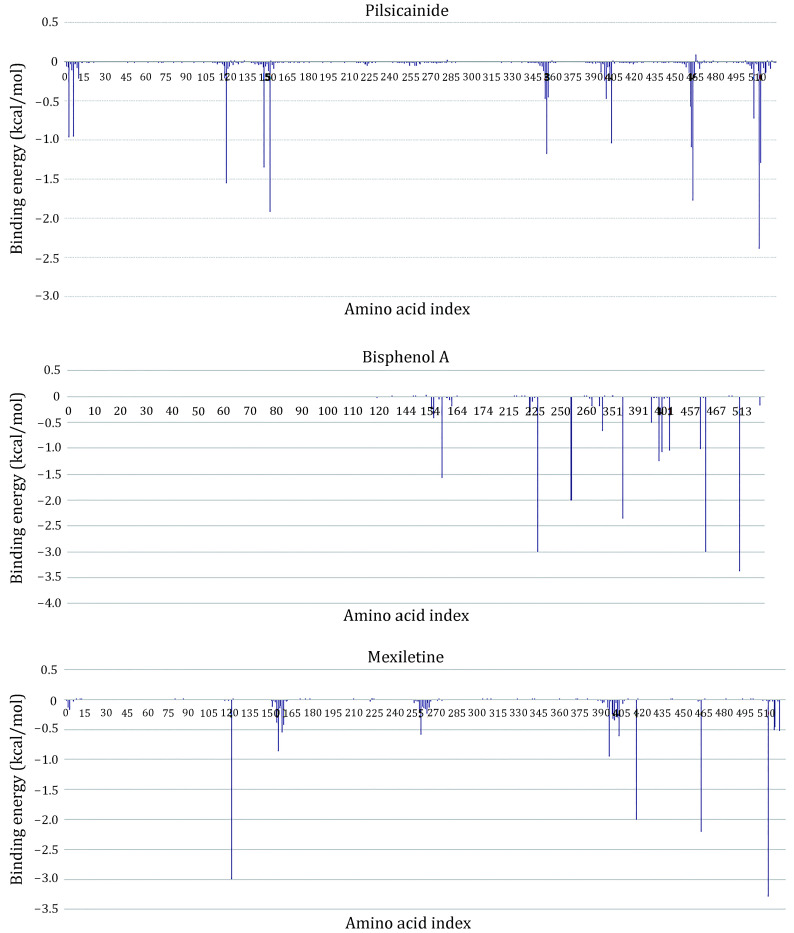
Residue interaction spectrum between the closed10 and three closed-state LAs. The horizontal axis is amino acid index and the vertical axis is the binding energy

**Figure 7:**
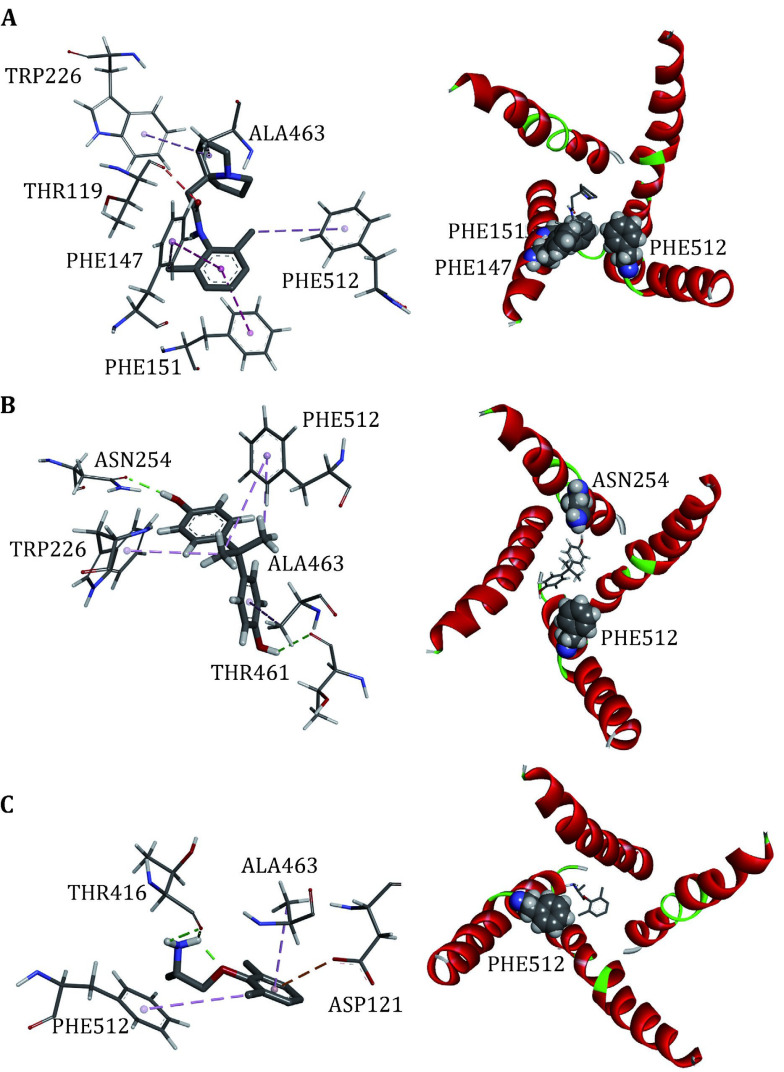
Interactions between Na_v_1.5 PD and LAs, Pilsicainide (**A**), Bisphenol A (**B**) and Mexiletine (**C**). The interaction diagrams (left column) were calculated and all of the structures were generated by using Discovery Studio client (version 4.1). S6s in four domains (right column) are shown in cartoon and the binding sites are shown in sphere, colored by spectrum

To further investigate the binding sites of LAs, the binding energies are decomposed to each residue (Fig. 6). For Mexletine, the interaction energy of D121 (−3.0035 kcal/mol), T416 (−2.001 kcal/mol), A463 (−2.202 kcal/mol) and F512 (−3.29 kcal/mol) are less than −2.0 kcal/mol. Of these four residues, the contribution of F512 (F1760 in Na_v_1.5α) is the most significant, which is consistent with the previous report that this residue is the key residue of LA binding with Na_v_1.5. According to the analysis of the interaction type (Fig. 7C), the interaction between F512 and Mexletine is mainly through the pi-alkyl interaction.

For Bisphenol A, the residues T226, N254, F355, T461, A463 and F512 play major role in the interaction between Bisphenol A and closed10. At the same time, we can see that Bisphenol A interacts with only F512 but not with Y519 (Y1767 in Na_v_1.5α). Previous researches have shown that Y1767 played a vital role in the use of state-dependent inhibition for LA, and there was no significant effect in the tonic block. F512 is the same binding site of Bisphenol A and Mexletine.

Pilsicainide is the weakest inhibitor among the three closed-state LAs. The main sites of action are T119, F147, F151, W226, A463 and F512. Among these critical sites, only the interaction energy of PHE512 is lower than −2.0 kcal/mol, which contributes the highest binding energy to all residues. Although no experiments have been made to point out whether Pilsicainide has direct interaction with F512, it is an important binding site for LAs. We have reason to speculate that, similar to Bisphenol A and Mexletine, Pilsicainide also interacts with the F512.

## CONCLUSION

In this work, the pore domain of voltage gated sodium channel Na_v_1.5 in closed-state was constructed. The diameter of the final structure in the region of the activation gate is 3.48 Å and is less than the diameter of the hydrated sodium ion. It cannot allow the passage of sodium ions and demonstrates that the model we have built is in closed-state. Furthermore, the interactions between the model and the three closed-state LAs were also studied and the results further validated the reasonability of the built model. Our results may provide a reference for studying the mechanism of interaction between Na_v_1.5 and LAs and may also provide a theoretical basis for further screening and design of drugs.

## METHOD

### Prediction of contacts between residues

Recently it was shown that the residue–residue contacts might be determined by co-evolutionary residue pairs, *i*.*e*., if two residues in a protein sequence or in two different sequences form direct interaction or contact in 3D structure, they usually co-evolve (Jarzynski 2012; Lunt *et al*. 2010; Morcos *et al*. 2011, 2014; Weigt *et al*. 2009). DCA calculates a score for each residue pair in a sequence and the score can be used to evaluate if the two residues in the pair can form contact. The detail of DCA has been described in many papers (Jarzynski 2012; Lunt *et al*. 2010; Morcos *et al*. 2011, 2014; Weigt *et al*. 2009) and will not be explained here.

DCA uses aligned homologous sequences of the target sequence to analyze the co-evolutionary residues and calculates the score of evolutionary couplings (EC score) between two residues in the target sequence. The sequences of the four domains (DI–DIV) that form the Na_v_1.5 PD were obtained from NCBI (http://www.ncbi.nlm.nih.gov/) and each of them or each two of them were used as the target sequence(s) to calculate the EC score. The DCA was performed on EVcouplings online server (Aurell and Ekeberg 2012; Hopf *et al*. 2014; Marks *et al*. 2011; Morcos *et al*. 2011) and by using a pseudo-likelihood maximization (PLM) approximation (Ekeberg *et al*. 2012; Morcos *et al*. 2011). The search tool HHblits (Remmert *et al*. 2011; Vikram *et al*. 2016) was used to generate multiple sequence alignment. In order to assess whether the EC scores can be used to determine residue–residue contacts within and between protein domains, we built an evaluation set. This evaluation set includes the Na_v_ channels with known structures. We calculated the EC scores and distances between each residue pair. The distance of a pair of residues is defined as the shortest atom distance between the two residues.

### Structural modeling and selection

Using the ROSETTA-membrane modeling protocol (version 3.4) (Sidhartha *et al*. 2010; Subbotina *et al*. 2010), the Homology, *de novo*, and full-atom modeling of the four domains of the PD were built by the crystal structure of Na_v_PaS (PDB ID: 5X0M) as template. Models with lower energy and higher contact coincidence were selected. The energy was calculated by rosettaMP (Alford *et al*. 2015) and the contact coincidence was defined by the ratio of the predicted contacts and the true contacts of the intra-domains. Then, these selected models were aligned to the corresponding domains of their templates by a structural alignment algorithm TM-align (Zhang and Skolnick 2005) to assemble them to form candidates of the PD structure. The energies of these assembled structures were then evaluated by using rosettaMP and the residue–residue contacts between two domains were analyzed by DCA. Similar to the selection strategy for the monomers or domain structures, the structures with lower energy and higher contact coincidence were selected. Here the contact coincidence was the ratio of the predicted contacts and the true contacts between two domains. The selected structure was kept for further optimization and analysis.

### Molecular dynamical simulations and docking

**Figure 8:**
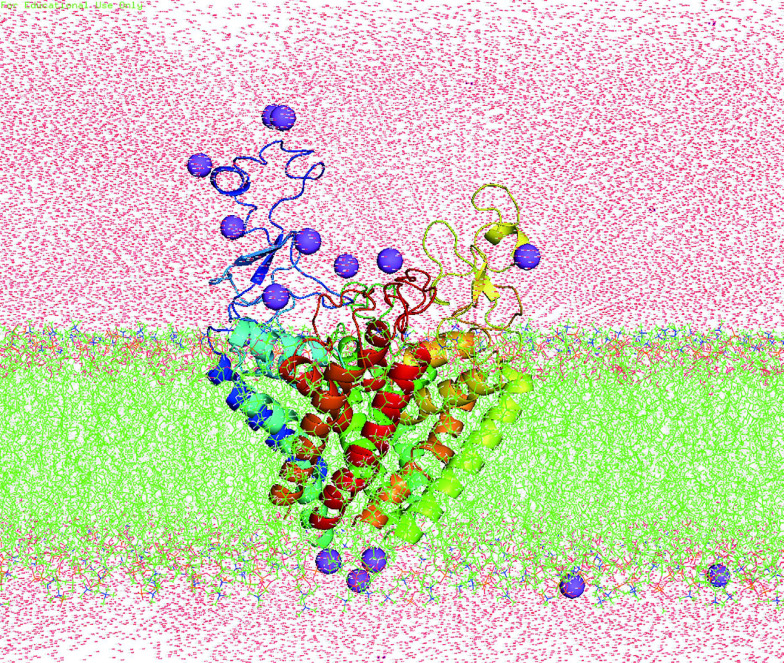
System used for MD simulations of Na_v_1.5 PD. Na_v_1.5 PD (in spectrum cartoon) embedded in a POPC lipid bilayer (in green stick). Sodium ions are purple

All Molecular dynamical (MD) simulations were done by the software package Amber16 (Case *et al*. 2016). The setting of parameters and MD process were nearly the same as our previous work (Ji *et al*. 2018) and only briefly described in the following: The modeled structure was oriented by using the PPM server (Lomize *et al*. 2011) along *Z*-axis and then embedded in a homogeneous palmitoyloleoyl phosphatidylcholine (POPC) bilayer and solvated by 20 Å water molecules on both sides of the membranes (Fig.8) by using Charmm-GUI (Lee *et al*. 2015). 0.15 mol NaCl was added to neutralize the system. The final system contained 166,700 atoms, including 8316 protein atoms, 444 POPC lipid molecules, 2889 water molecules, 13 Na^+^ ions. After 50,000 steps of optimization (25,000 cycles of steepest descent and 25,000 cycles of conjugate gradient minimization), the system was gradually heated from 0 to 300 K by Langevin thermostat in NVT ensemble. During the equilibration process, the protein was firstly fixed while water and lipid were allowed to equilibrate for 2 ns. Then the contraction of predicted contact residue pairs was restrained by a harmonic potential with force constant reducing gradually from 10 to 0.1 kcal/mol in four 10-ns running steps. Finally, the formal simulations were carried out for a total of 400 ns at constant temperature (300 K) and pressure (1 bar) conditions, which are maintained by using the periodic boundary conditions of NPT ensemble. The module of cpptraj in Amber16 software package was used to analyze the resulting trajectories and the code MOLE 2.0 (Sehnal *et al*. 2013) was used to calculate the pore radius.

For the docking calculations, three closed-state LA drugs (Pilsicainide, Bisphenol A and Mexiletine) were downloaded from the ChemSpider (http://www.chemspider.com/). These compounds are sorted in supplementary Table S2 according to their *K*_d_ values. In this work, Autodock4.2 was employed for docking calculations and Autodock Tools (ADT) (Morris *et al*. 2009) was used for preparing molecules and all of the hydrogens were added by using REDUCE (Word *et al*. 1999). The docking protocol is similar with the previous literature on ligand docking to Na_v_ channels (Lorena *et al*. 2016). Modeled structures were defined as receptors and the coordinates of AutoGrid center were determined by the center of the largest binding pocket, which was generated from receptor cavities. The grid size was set to 100 × 100 × 100 points with grid spacing of 10 Å. The grid box included almost the entire channel residues of the receptor. For drug molecules, they were defined as ligands. The procedure was carried out by keeping the protein rigid and the docked ligands were considered flexible. All of the docking decoys were clustered with cutoff 2 according to the root mean square deviation (RMSD) and the lowest-energy docked structures from each cluster were selected as the models of the docked ligand (MDL). Finally, the conformation with the lowest energy was selected as the complex structure for further analysis.

### MM-GBSA binding-energy calculations

The selected docking poses of three LA_Na_v_1.5 complexes were used as the initial structures for MD simulation. Topology and parameter files for ligands were generated using the antechamber program (Wang *et al*. 2006) in the Amber16 package. Partial charges of ligands were calculated using the AM1-BCC method (Araz 2002). Each LA_Na_v_1.5 complexes was immersed in a 20 Å^3^-sized cubic box of TIP3P water molecules, and the parameters for amino acids and ligand molecules were assigned using the AMBER-FF14SB force field (Maier *et al*. 2015) and the GAFF force field, respectively. The calculation steps and parameter setup were the same as those for Na_v_1.5_PD mentioned above. For each LA_Na_v_1.5 system, binding energies were computed using the popular Molecular mechanics/Generalized Born Surface Area (MM-GBSA) module of AMBER after equilibrium and using a 1-ps frame separation interval.

According to the MM-GBSA protocol, the free binding energy estimation (Δ*G*_bind_) can be calculated as follow:

\begin{equation*}\begin{split} 
\Delta {G_{{\rm{bind}}}} \approx &\Delta {E_{{\rm{gas}}}} + \Delta {G_{{\rm{sol}}}} - T\Delta S = \Delta {E_{{\rm{ele}}}} + \Delta {E_{{\rm{vdW}}}}\\& + \Delta {G_{{\rm{GB}}}} + \Delta {G_{{\rm{surf}}}} - T\Delta S
\end{split}\end{equation*}

In the above equation, Δ*G*_bind_ is decomposed into different energy terms. Because the structures of LA_Na_v_1.5 complex, receptor Na_v_1.5, and ligand LAs are extracted from the same trajectory, the internal energy change is canceled. Thus, the gas phase interaction energy (Δ*E*_gas_) between Na_v_1.5 and LA is the sum of electrostatic binding energies (Δ*E*_ele_) and the estimate of the lipophilic van der Waals interaction energy (Δ*E*_vdW_) between the Na_v_1.5 and LA. The solvation free energy (Δ*G*_sol_) is formed by the polar and non-polar energy terms. The polar solvation energy (Δ*G*_GB_) was calculated by using GB model. The non-polar contribution was calculated based on the solvent accessible surface area (Δ*G*_surf_).

## Conflict of interest

Xiaofeng Ji, Yanzhao Huang and Jun Sheng declare that they have no conflict of interest.
